# Antiviral and Anti-Inflammatory Activities of Fluoxetine in a SARS-CoV-2 Infection Mouse Model

**DOI:** 10.1192/j.eurpsy.2023.318

**Published:** 2023-07-19

**Authors:** D. Péricat, S. A. Leon-Icaza, M. Sánchez-Rico, C. Mühle, I. Zoicas, F. Schumacher, R. Planès, R. Mazars, G. Gros, A. Carpinteiro, K. A. Becker, J. Izopet, N. Strub-Wourgaft, P. Sjö, O. Neyrolles, B. Kleuser, F. Limosin, E. Gulbins, J. Kornhuber, E. Meunier, N. Hoertel, C. Cougoule

**Affiliations:** 1 Institute of Pharmacology and Structural Biology (IPBS), Toulouse; 2 Université Paris Cité, Paris, France; 3 Friedrich-Alexander-University of Erlangen-Nuremberg, Erlangen; 4 Freie Universität Berlin, Berlin; 5 Institute for Molecular Biology, University Medicine Essen, Essen, Germany; 6 Toulouse Institute for Infectious and Inflammatory Diseases (INFINITy), Toulouse, France; 7 Drugs for Neglected Diseases Initiative, Geneva, Switzerland

## Abstract

**Introduction:**

The coronavirus disease 2019 (COVID-19) pandemic continues to cause significant morbidity and mortality worldwide. Since a large portion of the world’s population is currently unvaccinated or incompletely vaccinated and has limited access to approved treatments against COVID-19, there is an urgent need to continue research on treatment options, especially those at low cost and which are immediately available to patients, particularly in low- and middle-income countries. Prior in vitro and observational studies have shown that fluoxetine, possibly through its inhibitory effect on the acid sphingomyelinase/ceramide system, could be a promising antiviral and anti-inflammatory treatment against COVID-19.

**Objectives:**

The aim of this sudy was to test the potential antiviral and anti-inflammatory activities of fluoxetine against SARS-CoV-2 in a K18-hACE2 mouse model of infection, and against several variants of concern in vitro, and test the hypothesis of the implication of ceramides and/or their derivatives hexosylceramides.

**Methods:**

We evaluated the potential antiviral and anti-inflammatory activities of fluoxetine in a K18-hACE2 mouse model of SARS-CoV-2 infection, and against variants of concern in vitro, i.e., SARS-CoV-2 ancestral strain, Alpha B.1.1.7, Gamma P1, Delta B1.617 and Omicron BA.5.

**Results:**

Fluoxetine, administrated after SARS-CoV-2 infection, significantly reduced lung tissue viral titres (Figure 1) and expression of several inflammatory markers (i.e., IL-6, TNFα, CCL2 and CXCL10) (Figure 2). It also inhibited the replication of all variants of concern *in* vitro. A modulation of the ceramide system in the lung tissues, as reflected by the increase in the ratio HexCer 16:0/Cer 16:0 in fluoxetine-treated mice, may contribute to explain these effects (Figure 3).

**Image:**

**Figure:**
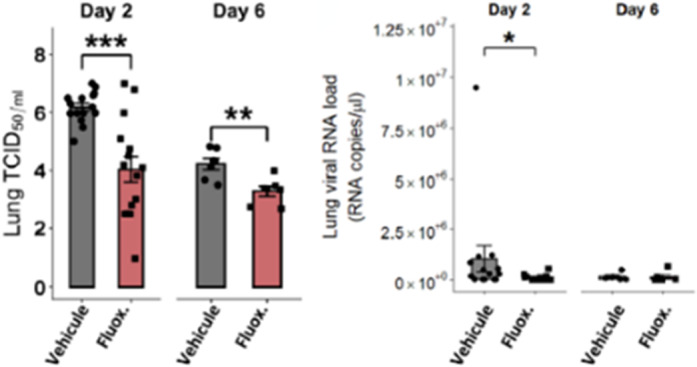

**Image 2:**

**Figure:**
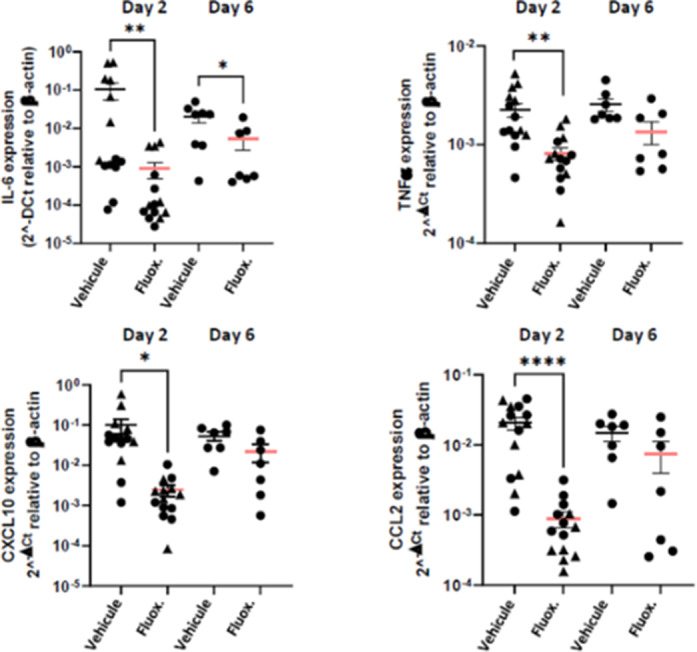

**Image 3:**

**Figure:**
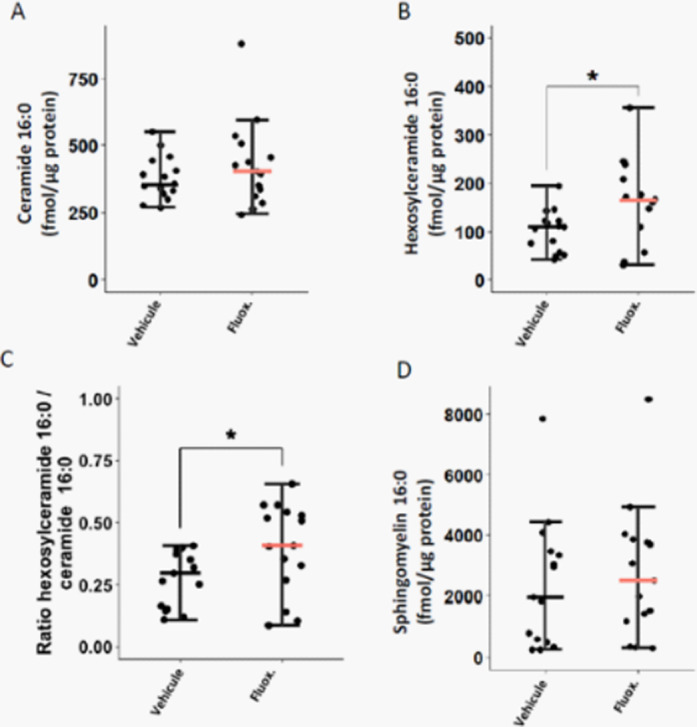

**Conclusions:**

Our findings demonstrate the antiviral and anti-inflammatory properties of fluoxetine in a K18-hACE2 mouse model of SARS-CoV-2 infection, and its in vitro antiviral activity against variants of concern, establishing fluoxetine as a very promising candidate for the prevention and treatment of SARS-CoV-2 infection and disease pathogenesis.

**Disclosure of Interest:**

None Declared

